# PDZ binding kinase (PBK) is a theranostic target for nasopharyngeal carcinoma: driving tumor growth via ROS signaling and correlating with patient survival

**DOI:** 10.18632/oncotarget.8445

**Published:** 2016-03-28

**Authors:** Meng-Yao Wang, Zhi-Rui Lin, Yun Cao, Li-Sheng Zheng, Li-Xia Peng, Rui Sun, Dong-Fang Meng, Ping Xie, Jun-Ping Yang, Li Cao, Liang Xu, Bi-Jun Huang, Chao-Nan Qian

**Affiliations:** ^1^ State Key Laboratory of Oncology in South China, Collaborative Innovation Center for Cancer Medicine, Sun Yat-sen University Cancer Center, Guangzhou, Guangdong, China; ^2^ Department of Nasopharyngeal Carcinoma, Sun Yat-sen University Cancer Center, Guangzhou, Guangdong, China; ^3^ Key Laboratory of Medical Reprogramming Technology, Shenzhen Second People's Hospital, the First Affiliated Hospital of Shenzhen University, Shenzhen, China; ^4^ Department of Pathology, Sun Yat-sen University Cancer Center, Guangzhou, Guangdong, China; ^5^ Department of Pharmacy, Sun Yat-sen University Cancer Center, Guangzhou, Guangdong, China

**Keywords:** PBK/TOPK, nasopharyngeal carcinoma, ROS, JNK/P38 pathway

## Abstract

Nasopharyngeal carcinoma (NPC) is well known as one of the most common malignancies in southern China and Southeast Asia. However, the mechanisms underlying NPC progression remain poorly understood. Herein, through overlapping the differentially expressed genes from 3 microarray data sets with the human kinome, we identified PBK, a serine-threonine kinase, is highly upregulated and has not been intensively investigated in NPC. PBK was required for malignant phenotypes of NPC, as PBK depletion by RNAi and inhibition by specific inhibitor HI-TOPK-032 obviously reduced cell proliferation and xenograft tumor growth in mice. Moreover, we determined that targeting PBK could accelerate apoptosis by inducing ROS that activates JNK/p38 signaling pathway. In NPC patients, elevated PBK expression in primary tumor positively correlated to clinical severity such as advanced T stage, high death risk and disease progression, and it could serve as an unfavorable independent indicator of overall survival and disease-free survival. Altogether, our results indicate that PBK is a novel significant regulator of NPC progression and a potential therapeutic target for NPC patients.

## INTRODUCTION

Nasopharyngeal carcinoma (NPC) has a high incidence rate in southern China and Southeast Asia [1-3]. Radiotherapy and Platinum-based chemotherapy are standard treatment modalities for NPC [4-6]. Although many improvements in technology and equipment of radiotherapy have been achieved, the outcome of patients with locoregionally advanced NPC is still unsatisfactory. Locoregional relapse and distant metastasis are the main reason of treatment failure [7, 8]. The molecular mechanisms regulating NPC recurrence and metastasis are not fully understood.

It is well known that the deregulated expression or activity of many kinases play a pivotal role in tumor biology processes including uncontrolled proliferation, metastasis and angiogenesis. Since malignant tumors are adapted to dysregulation of kinases, it is compelling to target them to lead to dramatic clinical responses. To date, some of the kinase inhibitors have been successfully developed into cancer therapy, for instance, Imatinib to BCR-ABL in chronic myelogenous leukemia [9], vemurafenib [10] to BRAFV600E in melanoma, cetuximab to ErbB1 in metastatic colon cancer [11], sunitinib to PDGFR, KIT and FLT3 in kidney cancer [12], and zactima to EGFR, VEGFR and RET in thyroid carcinoma [13]. However, there is no well-accepted targeted drug for NPC so far, and the explorations of more druggable targets are in need to better improve NPC patient survival.

PBK/TOPK, a serine-threonine kinase, is highly expressed in various cancers such as lymphoma, leukemia, melanoma, colorectal, breast cancer, lung and glioma [14-19]. PBK is a mitogen-activated protein kinase (MAPKK) between MEK1/2 and MEK7 and can phosphorylates P38, JNK and ERK involving in many cellular functions [14, 20-23]. Zhu et al. demonstrated that positive feedback between TOPK and ERK2 promotes the tumorigenic properties of colorectal cancer cells [14]. PBK is also a mitosis kinase that it is activated by the cdk1/cyclin B1 complex to promote cytokinesis through phosphorylation of PRC1 [24-26]. Moreover, PBK has been recognized as a metastasis-promoting kinase that it promotes cell migration by modulating the PI3K/PTEN/AKT pathway in lung cancer [27] and is highly expressed in circulating tumor cells, enabling metastasis of prostate cancer [28]. Remarkably, a preceding report shows that PBK includes in the “consensus stemness ranking signature” gene list that is up-regulated in cancer stem cell enriched tumors [29].

Further, a specific inhibitor HI-TOPK-032 [30] for PBK/TOPK reduces cell viability and colony formation via a dramatic increase in apoptotic cells *in vitro* and results in a significant decrease of tumor growth *in vivo*. Then, through the normal tissues data published recently [31] and literature mining [17], we put our intention on PBK/TOPK because it is hard to detect in vital organs except for testis and might be a promising molecular target.

## RESULTS

### PBK expression is elevated in NPC tissues and cell lines

The human kinome [35], containing about 518 kinase-coding genes, prominently serves as key regulators in signal transduction and orchestration of complex cellular processes via adding phosphate groups to substrate proteins. As mentioned before, the treatment of NPC runs into the choke point. Whole-exome and targeted deep sequencing of 128 NPC cases found mutations affecting 54 kinase genes, suggesting that kinase-driven pathways can be crucial in NPC progression [36]. Likewise, we suppose that nonmutant kinases with global changes in the expression may contribute to NPC development.

**Figure 1:**
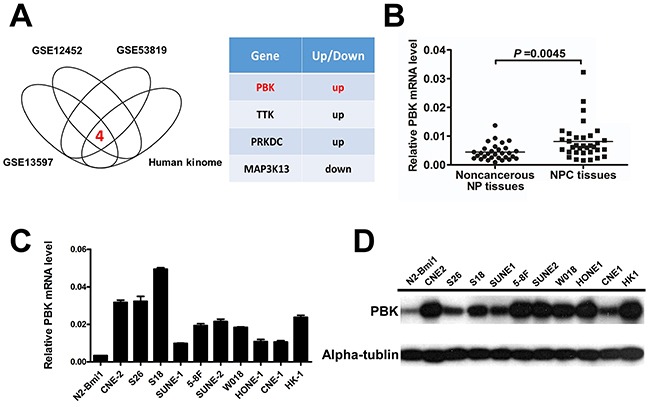
PBK/TOPK expression is frequently upregulated in NPC tissues and cell lines **A.** PBK was identified to be a potential oncogene in NPC from analysis in microarray data and the human kinome. **B.** The mRNA levels of PBK (normalized to β-actin) in NPC tissues and noncancerous NP tissues were confirmed by quantitative real-time PCR, showing that PBK expression was significantly higher in NPC tissues than in NP tissues. P values were calculated using the Student t test. The relative PBK mRNA levels and protein levels were determined by quantitative real-time PCR **C.** and immunoblotting **D.** showing that PBK is differentially expressed in NPC cell lines and N2-bmi1 immortalized nasopharyngeal epithelial cells. Column, mean; error bar, ± SD (from triplicates).

Thus, we overlap the differently expressed genes from 3 microarray data set (GSE12452 [37, 38], GSE53819 [33], GSE13597 [39]) with the human kinome, and found that there are 4 differently expressed kinase genes (PBK, TTK, PRKDC, MAP3K13) (Figure 1A). Subsequently, PBK mRNA expression was measured in 34 NPC patient tissue samples and 30 non-cancerous nasopharyngeal tissues by real —time quantitative PCR (qPCR). The relative expression level of PBK was significantly higher in NPC tissues compared with the NP tissues (Figure 1B, P =0.0045, student t test). Increased PBK mRNA and protein expression were also observed in NPC cell lines compared with the N2-bmi1 immortalized nasopharyngeal epithelial cells (Figure 1C and 1D). Therefore, we hypothesized that PBK plays a critical role in NPC tumorigenesis and progression.

### Suppression of PBK inhibits NPC cell growth *in vitro*

**Figure 2:**
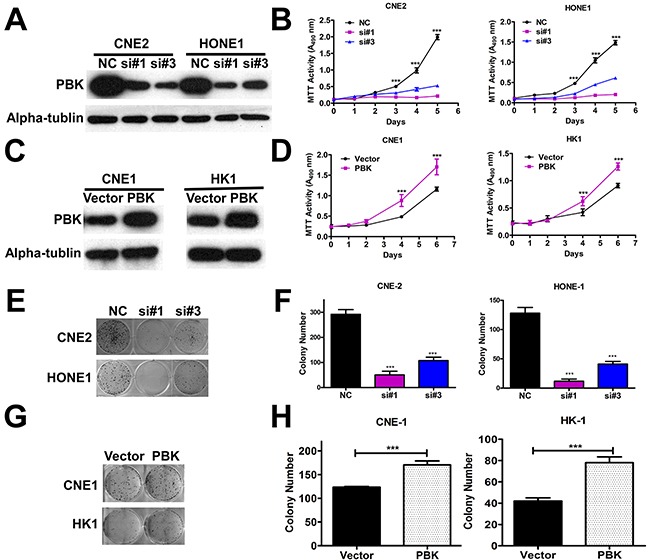
Regulation of PBK expression affects growth in NPC cells *in vitro* **A and C.** Transient suppression and overexpression of PBK in NPC cells were determined by immunoblotting analysis, a-tubulin was used as a loading control. **B and D.** Cell proliferation was determined by the MTT assay; ***P < 0.001, Student t test. Colony formation ability in suppression of PBK; Representative micrographs **E.** and quantification **F.** of crystal violet stained cells from 3 independent experiments; Overexpression of PBK increased colony cell numbers **G and H.** ***P < 0.001, Student t test. The data are presented as the mean ± SD (from triplicates).

To explore the role of PBK in NPC cell growth, we transfected CNE-2 and HONE-1 cells with siRNA (PBKsi#1 and #3) or negative control siRNA. The siRNA suppression efficiency of PBK protein levels was confirmed by immunoblotting (Figure 2A). We observed that PBK suppression significantly inhibited NPC cell proliferation (Figure 2B) and colony formation ability (Figure 2E and 2F). Meanwhile, we also generated cell lines (CNE-1 and HK-1 with poor colony formation ability *in vitro*) overexpressing PBK. PBK protein expression was validated by immunoblotting (Figure 2C). Overexpression of PBK in CNE-1 and HK-1 cells resulted in increased cell proliferation (Figure 2D) and colony formation (Figure 2G and 2H).

### PBK promotes NPC cell growth *in vivo*

**Figure 3:**
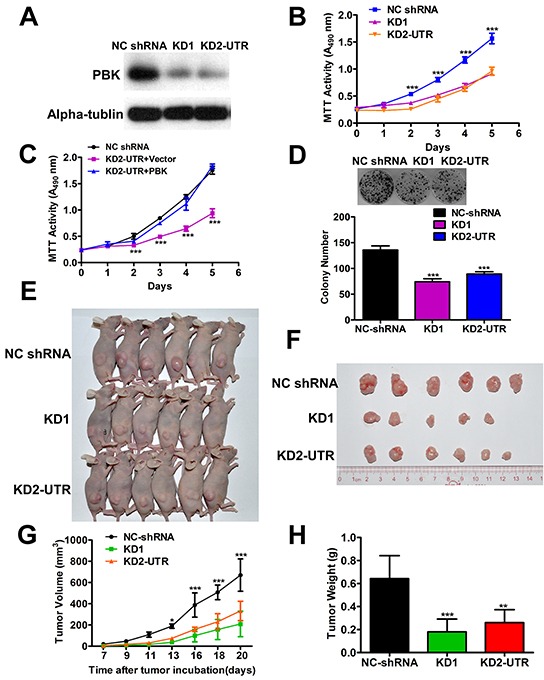
Suppression of PBK inhibits the growth of NPC cells *in vivo* **A.** Stable suppression of PBK in NPC cells were determined by immunoblotting, α-tubulin was used as a loading control. **B.** Cell proliferation was determined by the MTT assay; ***P < 0.001, Student t test. Colony formation ability, representative micrographs and quantification **D.** of crystal violet stained cells from 3 independent experiments; ***P < 0.001, Student t test. **C.** Cell growth was rescued in PBK knockdown cell by overexpressing PBK. **E.** The PBK knockdown CNE2 (KD1 and KD2-UTR) cells and the negative control shRNA cells were subcutaneously injected into nude mice. **F.** The photographs of isolated tumors. **G.** The growth curve indicates CNE2 growth suppression upon PBK knockdown *in vivo*. **H.** The terminal tumor weights are also decreased compared with the negative control. *P < 0.05, **P < 0.01, ***P < 0.001, Student t test. The results are presented as the mean ± SD, n = 6 per group. Scale bar, 1 cm.

To further explore that PBK is crucial for NPC growth *in vivo*, we established CNE-2 cell stably expressing PBK-targeting shRNA using 2 different shRNA (PBK KD1 and KD2-UTR) or a non-target shRNA in CNE-2 cells. The shRNA suppression efficiency of PBK protein levels was validated by immunoblotting (Figure 3A). We observed that PBK knockdown (PBK sh#2 and #4) effectively impeded NPC cell growth *in vitro* (Figure 3B), the same effect on the colony formation abilities (Figure 3D). Overexpression of PBK in CNE2 stably expressing KD2-UTR cell, the cell growth was rescued, compared with the PBK knockdown cell transfected with vector (Figure 3C), indicting PBK plays an important role in NPC cell growth. Then, we utilized an animal xenograft model, CNE-2-shRNA (PBK KD1 and KD2) and negative control shRNA cells were subcutaneously injected into nude mice. CNE-2-shRNA cells group presented decreased tumor growth and weight compared with control group (Figures 3G and 3H). The photographs of isolated tumors are shown (Figure 3F), respectively.

### Oxidative stress induced by HI-TOPK-032 promotes cell apoptosis through activating MAPK signaling pathway

**Figure 4:**
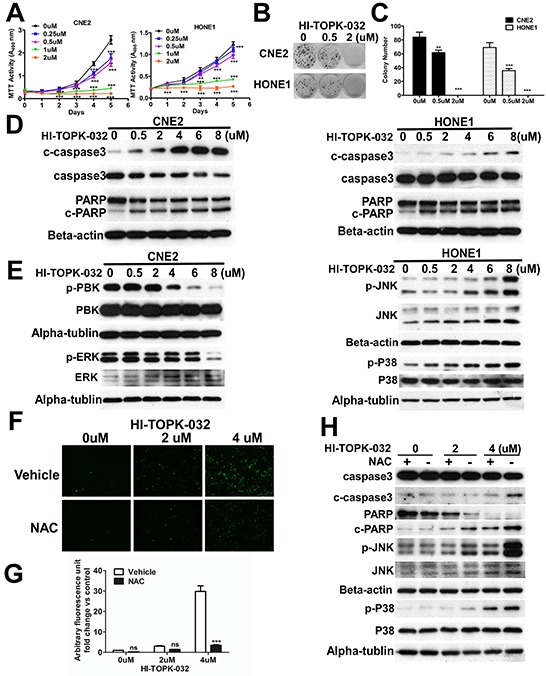
Oxidative stress induced by HI-TOPK-032 promotes apoptosis via the MAPK signaling pathway **A.** HI-TOPK-032 inhibits NPC cell growth in a dose-dependent manner. Cells were treated with HI-TOPK-032 and proliferation was measured by MTT assay. **P < 0.01, ***P < 0.001, Student t test. Colony formation ability; Representative micrographs **B.** and quantification **C.** of crystal violet stained cells from 3 independent experiments; ***P < 0.001, Student t test. **D.** HI-TOPK-032 induces NPC cell apoptosis in a dose-dependent manner. **E.** Effect of HI-TOPK-032 on MAPK signaling pathway in NPC cells. Cells were treated with HI-TOPK-032 for 48 hours in medium containing 10% FBS and analyzed by immunoblotting analysis. Similar results were observed from 2 independent experiments. **F.** ROS levels as indicated by CM-H2DCFDA in NPC cells treated with HI-TOPK-032 in 0μM, 2μM and 4μM for 6 hr in the presence or absence of NAC. A representative picture from multiple fields is shown for each treatment. Fluorescence signals are quantified **G.** Photomicrographs are 100×. ***P < 0.001, Student t test. The data are presented as the mean ± SD (from triplicates). **H.** Immunoblotting of apoptosis marker and MAPK signaling pathway from NPC cells treated with increasing concentrations of HI-TOPK-032 for 48hr in the presence or absence of NAC.

To examine the effect of HI-TOPK-032 on NPC cell proliferation, we performed the MTT assay. After treatment with HI-TOPK-032, results indicated that NPC cell growth was strongly suppressed by HI-TOPK- 032 in a dose-dependent manner (Figure 4A), and the same result was observed in colony formation abilities (Figures 4B and 4C). Next, to examine the effect of HI-TOPK-032 on apoptosis, CNE-2 and HONE-1 cells were treated with HI-TOPK-032 and then incubated for 48 hours. Results showed that hallmarks of apoptosis such as induction of caspase-3 cleavage and PARP cleavage in a dose-dependent manner (Figure 4D), indicating that targeting PBK can inhibit NPC cell growth by inducing cell apoptosis.

To further study, we therefore explored whether targeting PBK can have an effect on the regulation of MAPK signaling pathway. HI-TOPK-032 directly suppresses PBK kinase activity, meanwhile, the enhanced phosphorylation of JNK and P38 were detected by immunoblotting in a dose-dependent manner (Figure 4E), indicating that HI-TOPK-032 promotes apoptosis via activating JNK and P38. The increase of intracellular ROS levels in response to HI-TOPK-032 treatment led us to examine the relationship between the inactivation of PBK, oxidative stress and MAPK signaling pathway. We found that targeting PBK effectively induced the production of ROS (Figure 4F). Interestingly, co-treatment with the antioxidant NAC was able to repress the induction of ROS by HI-TOPK-032(Figure 4F), suggesting that inactivation of PBK leads to accumulation of intracellular ROS. The data showed that co-treatment with the antioxidant NAC was able to abrogate the stimulatory effects of HI-TOPK-032 on JNK and P38, and the same results in induction of caspase-3 cleavage and PARP cleavage (Figure 4H), indicating that targeting PBK by HI-TOPK-032 induces oxidative stress via MAPK signaling pathway.

### HI-TOPK-032 inhibits tumor growth *in vivo*

**Figure 5:**
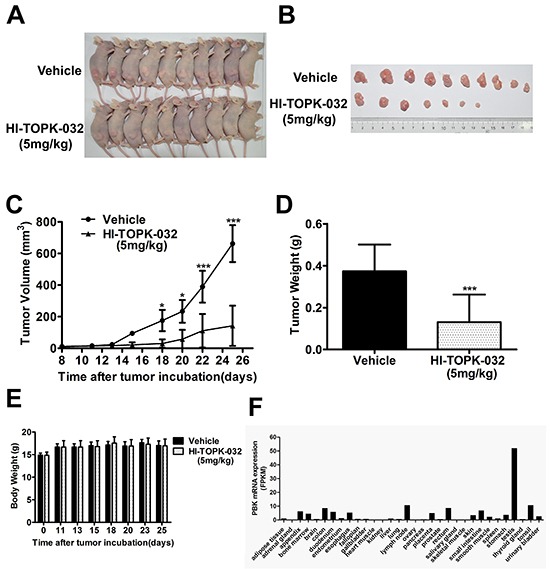
HI-TOPK-032 prevents xenograft tumor growth **A.** CNE2 cells were subcutaneously transplanted into the dorsal right flank of mice nude mice. After establishment of tumors, mice were treated with HI-TOPK-032 (5 mg/kg) or vehicle three times a week. **B.** The tumors were extracted after 14 days of treatment and a representative tumor from each group is shown. **C.** Mean tumor volume was calculated. Mean tumor volumes and tumor weights of HI-TOPK-032 treated group (5 mg/kg) were significantly smaller than the vehicle control group **D.** ***P < 0.001, Student t test. Mice in both treated and untreated groups were obtained in every injection. ***P < 0.001, Student t test. **E.** Body weights of vehicle- or HI-TOPK-032-treated mice throughout the treatment. HI-TOPK-032 had no effect on mouse body weight. The results are presented as the mean ± SD, n = 10 per group. **F.** PBK mRNA level in normal tissues from literature data mining.

To further evaluate the anti-tumor potential of HI-TOPK-032 *in vivo*, CNE-2 cells were injected into the dorsal right flank of athymic nude mice. After establishment of tumors, an HI-TOPK-032-treated group (n = 10) received an injection of the compound (5 mg/kg) three times a week for 14 days, whereas a control group (n = 10) was injected with physiological saline. Treatment of mice with 5 mg/kg group presented significantly decreased tumor volume and weight compared with vehicle-treated group (Figure 5C and 5D). In addition, there were no overt signs of toxicity or significant loss of body weight similar to the vehicle-treated group (Figure 5E). The data showed that expression of PBK in 32 human organs tissues, PBK mRNA levels in vital organs is very low, such as brain, liver, kidney and lung, indicating that targeting PBK may be a useful and low-toxicity therapy.

### High-level PBK expression is an independent, unfavorable prognostic indicator for NPC

**Figure 6:**
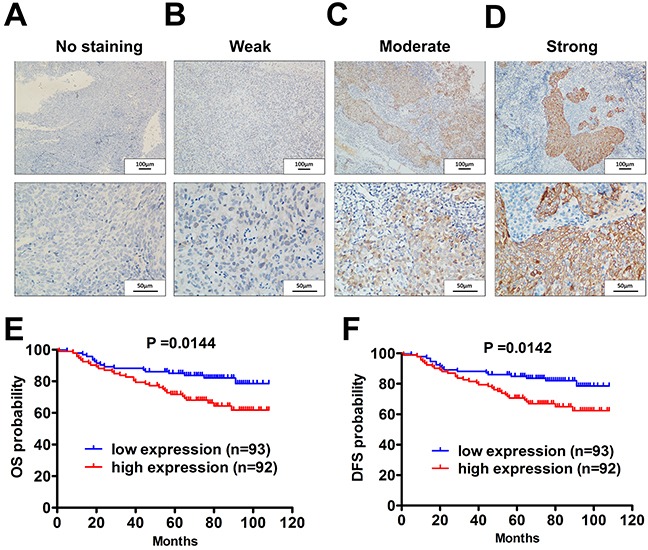
Elevated PBK/TOPK level correlates with shorter overall survival and disease free survival in NPC patients **A-D.** Levels of PBK protein expression in NPC tissues are shown under both low and high magnifications of a light microscope. Scale bars, 100μm and 50μm. **E.** The overall survival (OS) rate was significantly higher in the low PBK group. **F.** The disease-free survival (DFS) rate was also significantly higher in the low-PBK group.

Finally, we further evaluated the clinical implications of PBK expression using IHC staining in 185 NPC samples. PBK expression in NPC tissues were shown (Figure 6A-D). The correlations between PBK expression and clinicopathological characteristics are presented (Table 1). A high level of PBK in primary tumors was significantly correlated with the advanced T stage, high death risk and disease progression. Multivariate analyses of different prognostic parameters revealed that high PBK expression was an independent, unfavorable prognostic indicator for overall survival and disease free survival (Table 2). In the Kaplan—Meier analysis, OS and DFS was longer for patients with low PBK expression than those with high PBK expression (Figure 6E and 6F). Taken together, these analyses revealed that high PBK level in NPC significantly correlated with poor patient outcomes.

**Table 1:** Association between expression of PBK/TOPK and clinical characteristics in 185 NPC patients

Characteristics	No.	PBK/TOPK expression		P value (Chi-square test)
		Low	High	
Gender				
Male	142	73	69	0.574
Female	43	20	23	
Age				
<45	96	48	48	0.939
≥45	89	45	44	
T stage				
T1-2	56	35	21	**0.028**
T3-4	129	58	71	
N stage				
N0-1	104	57	47	0.162
N2-3	81	36	45	
M stage				
M0	169	89	80	0.838
M1	16	8	8	
Clinical stage				
I-II	22	14	8	0.182
III-IV	163	79	84	
Death				
Yes	49	17	32	**0.011**
No	136	76	60	
Disease progression				
Yes	24	6	18	**0.008**
No	161	87	74	
WHO histological classification Type 2				
Differentiated	15	8	7	0.942
Undifferentiated	169	89	81	

Abbreviations: WHO, World Health Organization

**Table 2:** Univariate and multivariate analysis of different prognostic parameters in NPC patients

Variables	Univariate analysis			Multivariate analysis		
	HR	Cl	p	HR	Cl	p
Gender	0.775	0.387-1.554	0.473	…	…	…
Age	2.123	1.187-3.798	**0.011**	2.472	1.356-4.504	**0.003**
T stage	2.178	1.056-4.495	**0.035**	1.640	0.780-3.448	0.192
N stage	1.268	0.724-2.221	0.406	…	…	…
M stage	7.289	3.739-14.213	**<0.001**	9.670	4.766-19.619	**<0.001**
Clinical stage	3.662	0.889-15.082	0.072	…	…	…
PBK/TOPK	2.051	1.139-3.694	**0.017**	2.182	1.190-4.003	**0.012**

## DISCUSSION

In the present study, we confirmed that PBK was a novel oncogene and a promising theranostic target for NPC. Our functional studies showed that knocking down of PBK expression suppresses cancer cell proliferation and colony formation in NPC cells, as well as HI-TOPK-032 treatment could induce a massive increase in ROS and activate P38/JNK pathways to promote apoptosis. In addition, elevated expression of PBK in NPC tissues associated with advanced T stage and disease progression. Moreover, overexpression of PBK was a significant independent prognostic factor of shortened overall survival and time to progression. Therefore, PBK should be explored further as a candidate target for molecular therapy in NPC.

Oxidative stress can significantly negatively impact cellular survival and lead to programmed cell death through apoptosis and autophagy [40-42]. Regulation of cell death and survival is also controlled in part by signaling cascade activated by the mitogen activated protein kinase (MAPK) [43, 44]. Our findings are consistent with studies in other cancers [45], suggesting that ROS accumulation subsequently induces prolonged c-Jun N-terminal kinase (JNK) cascade and P38MAPK cascade activation, then promotes cell apoptosis, however, the mechanisms of ROS inducing by HI-TOPK-032 need further investigation. In addition, we need to consider mechanisms of ROS promote persistent JNK/P38 activation, on one thing, ROS may inactivate inhibitors, which normally suppress JNK/P38 activation, therefore resulting in prolonged JNK/P38 activation. On the other thing, oxidized MKPs are rapidly degraded by the ubiquitin-proteasome pathway.

Our findings are similar to findings in other cancers [19, 30], who showed that the administration of HI-TOPK-032 at a dose of 5 mg/kg bodyweight as an experimental therapy led to a massive reduction in tumor volume, in the meantime, there were no obvious toxicity or significant loss of body weight similar to the vehicle-treated group, through the normal tissues data published recently [31], the data showed that expression of PBK in 32 human organs tissues, is hard to detect in vital organs except for testis and might be a promising molecular target

In summary, we found that PBK played a strong role in regulating NPC growth via activation of MAPK induced by accumulation of ROS in NPC cells, and PBK is a potential therapeutic targets for NPC treatment.

## MATERIALS AND METHODS

### Cell culture, cellular growth curve, and colony-formation assays

The human nasopharyngeal carcinoma cell lines CNE-2 and its clones(S18 and S26), Hone-1, SUNE-1 and its clone 5-8F, SUNE-2 and its clone W018, CNE-1, HK-1, were maintained in Dulbecco's modified Eagle's medium supplemented with 10% FBS at 37°C and 5% CO_2_.

Cellular growth curves were plotted by using the cellular viability values assessed by the MTT method (Cell Titer 96 Aqueous One Solution Cell Proliferation Assay solution; Sigma), as described previously [32]. Briefly, 1000 cells/200ml of medium were seeded into a 96-well plate (Corning) and cultured under normal conditions. At various time points after seeding, the cells in each well were stained with MTT (Sigma, M2128 ) for 3.5 h, the medium was discarded, and 200ml of DMSO was added to each well and incubated for 10 min, and the OD490 was determined with a microplate reader.

For the colony-formation assays, 500 cells/2ml were seeded into a 6-well plate (Corning). After 12 days, the cells were washed with phosphate-buffered saline (PBS), fixed with methanol for 10 min at room temperature, and stained with 1% crystal violet for 20 min. The colony was counted. All experiments were independently repeated at least three times.

### RNA isolation and real-time quantitative reverse-transcription PCR (qPCR)

Total RNA was extracted from cultured cell lines using Trizol reagent (Invitrogen) and subjected to reverse transcription using a cDNA Synthesis Kit (Thermo, K1622). Real-time qPCR was performed using a SYBR FAST Universal qPCR Kit (KAPA, KK4602). The relative expression levels of the target genes were calculated as two power values of ΔCt (the Ct of b-actin or PBK minus the Ct of the target gene). The sequences of the PCR primers used for amplification were as follows:

β-actin forward, 5′- AAGGTCATCC CTGAGCT GAA -3′;

β-actin reverse, 5′- TGACAAAGTG GTCGTTG AGG -3′;

PBK forward, 5′- GAAGAGGACTGAGAGTG GCT -3′;

PBK reverse, 5′- CTTCTGCATAAACGGAGA GGC -3′.

### Small interfering RNA transfection

The negative control small interfering RNA (NC) as purchased from GenePharma, and siRNA targeting human PBK are 5′- CCGCATGACT TTGACTGGAAT -3′(si#1) and 5′- GAGCTGGTGTCTGATTGTTAA -3′(si#3). Transient transfections of NPC cells were performed as described previously [33], by using the Lipofectamine RNAiMAX Reagent (Invitrogen) protocol with 60 pmol siRNA in Opti-MEM Medium (Invitrogen) were mixed, and incubated at room temperature for 5 min, then the mixture was added to the cells.

### Plasmid transfection experiment

Plasmid construction and transfection were performed as previously described [34]. Full-length human PBK cDNA were cloned into pcDNA3.1. CNE-1 and HK-1 cells seeded at 2 × 10^5^ cells per well of a 6-well,1mg plasmid DNA or the empty control vector plasmid were transfected using FuGENE HD transfection reagent (Roche) according to the manufacturer's instructions.

### Lentiviral transduction studies

Cell lines stably expressing PBK short hairpinRNA (shRNA) or a negative control shRNA were established by a BLOCK-iT Lentiviral Pol II miR RNAi system (Invitrogen) according to the manufacturer's instructions. The following are primers of PBK shRNA :

CDS-sh1:

5′-CCGGGGGAACTAGGCCACCTATTAACTCGAGTTAATAGGT

GGCCTAGTTCCCTTTTTG-3′

3′-AATTCAAAAAGGGAACTAGGCCACCTATTAACTCGAGTTAA

TAGGTGGCCTAGTTCCC-5′

CDS-sh2:

5′-CCGGCTCTTCTCTGTATGCACTAATCTCGAGATTAGTGCAT

ACAGAGAAGAGTTTTTG -3′

3′-AATTCAAAAACTCTTCTCTGTATGCACTAATCTCGAGATTAG

TGCATACAGAGAAGAG -5′

UTR-sh4:

5′-CCGGGAAGTGTGGCTTGCGTAAATACTCGAGTATTTACGC

AAGCCACACTTCTTTTTG -3′

3′-AATTCAAAAAGAAGTGTGGCTTGCGTAAATACTCGAGTATT

TACGCAAGCCACACTTC-5′

Lentiviruses were produced by 293T cells with one of the shRNA using FuGENE HD transfection reagent (Roche). Infectious lentiviruses were harvested 48h after transfection, and filtered through 0.45mm filter (Millipore, Bedford, MA). Cells were transduced with lentiviruses PBK shRNA or negative control shRNA, then cultured in medium containing 2mg/ml puromycin (Sigma) for 3 days to be used for selection and PBK knockdown efficiency were determined by immunoblotting.

### PBK/TOPK inhibitor

The PBK/TOPK inhibitor HI-TOPK-032 was purchased from sigma, and HI-TOPK-032 is determined to bind to the active site of PBK [30]. Stock concentration (2mg/ml) of HI-TOPK-032 was dissolved DMSO and warmed at 37°C. For the functional assays, cells were treated with the HI-TOPK-032 at a different concentration.

### Immunoblotting

Immunoblotting was performed as described previously [41, 45]. The sources of the primary antibodies were as follows: anti-PBK/TOPK(1:1,000), anti-phospho-PBK (1:1,000), anti-caspase3 (1:1,000), anti-cleaved-caspase3 (1:1,000), anti-PARP (1:1,000), anti- cleaved-PARP (1:1,000), anti-ERK1/2 (1:1,000), anti-phospho-ERK1/2 (1:1,000), anti-JNK (1:1,000), anti-phospho-JNK (1:1,000), anti-P38(1:1,000), anti-phospho-P38(1:1,000), and anti-a-tubulin(1:1,000) antibodies, anti-mouse and anti-rabbit peroxidase conjugated secondary antibodies were purchased from Cell Signaling Technology (Danvers, MA).

### Measurement of reactive oxygen species (ROS)

2×10^5^ cells /2ml were seeded into a 6-well plate (Corning) overnight, cells were pretreated with N-Acetyl-L-cysteine(NAC, sigma, A8199) or vehicle for 4h, then HI-TOPK-032 was added to the cells. After 6h cells were washed by PBS twice and loaded with 10mM general ROS indicatorCM-H2DCFDA (sigma) in serum free DMEM for 30 minutes at 37°C in the dark, then examined by fluorescence microscopy.

### Animal experiments

For the tumor xenograft experiments in suppression of PBK, female BALB/c athymic nude mice (3-4 weak of age) were randomly divided into three groups of 6 mice each, the cells (1 × 10^5^ cells/tumor in 150 mL DMEM with 25% matrigel) were subcutaneously injected into the right flanks of the nude mice. Tumor diameters were measured every 2 days after tumor were established. For the HI-TOPK-032 treatment: control group (n = 10), HI-TOPK-032 5 mg/kg of bodyweight (n = 10), Stock concentration (2mg/ml) of HI-TOPK-032 in DMSO was diluted in physiological saline. Tumor diameters were measured at each injection of HI-TOPK-032. All animal experiments were approved by the Institutional Animal Care and Use Committee of the Sun Yat-Sen University Cancer Center. Finally, the mice were euthanized, and the tumors were isolated and weighed, the tumor volume was calculated as volume = length × width^2^ × 0.5236.

### Human tissue samples

To compare the PBK levels among different stages of NPC development, 30 non-cancerous nasopharyngeal mucosa and 30 primary NPCs were obtained at the Department of Nasopharyngeal Carcinoma, Sun Yat-sen University cancer Center (SYSUCC). The PBK levels in the formalin-fixed paraffin- embedded tissue sections were measured by immune- histochemical analysis, as previously described (24). All these human tissue samples were obtained prior patient consents and the approval of the Institutional Clinical Ethics Review Board at SYSUCC. For each tumor, we determined a proportion score and an intensity score. The percentage of stained cells was categorized as no staining = 0, 1–10% of stained cells = 1, 11–50% = 2, 51–80% = 3, and 81–100% = 4. The intensity score represented the Cytoplasmic and membranous staining intensity staining intensity of the positive cells (0 = no staining; 1 = weak staining; 2= moderate staining; 3= strong staining).

### Statistical analysis

Student's t-test was used to compare two independent groups of data. One-way analysis of variance (ANOVA) was used to analyze the significance among multi-group. The median IHC staining score was used to divide the patients into low and high PBK expression groups. Chi-squared tests were applied to analyze the relationship between PBK expression and clinicopathological status. The significance of several variables for survival was analyzed using the Cox regression model in a multivariate analysis. P value < 0.05 was considered statistically significant in all cases.
